# A bi-directional Mendelian randomization study of the sarcopenia-related traits and osteoporosis

**DOI:** 10.18632/aging.204145

**Published:** 2022-07-02

**Authors:** Xue-Ying Ma, Hui-Min Liu, Wan-Qiang Lv, Chuan Qiu, Hong-Mei Xiao, Hong-Wen Deng

**Affiliations:** 1 Center for System Biology, Data Sciences, and Reproductive Health, School of Basic Medical Science, Central South University, Changsha, Hunan Province, P.R. China; 2 Tulane Center of Biomedical Informatics and Genomics, Deming Department of Medicine, School of Medicine, Tulane University, New Orleans, LA 70112, USA

**Keywords:** Mendelian randomization, sarcopenia, osteoporosis, fracture

## Abstract

Both sarcopenia and osteoporosis are common geriatric diseases causing huge socioeconomic burdens, and clinically, they often occur simultaneously. Observational studies have found a controversial correlation between sarcopenia and osteoporosis and their causal relationship is not clear. Therefore, we performed a bi-directional two-sample Mendelian randomization (MR) analysis to assess the potential causal relationship between sarcopenia-related traits (hand grip strength, lean mass, walking pace) and osteoporosis. Our analysis was performed by applying genetic variants obtained from the UK Biobank and the GEnetic Factors for OSteoporosis (GEFOS) datasets. We used inverse-variance weighted (IVW) and several sensitivity analyses to estimate and cross-validate the potential causal relationship in this study. We found that bone mineral density (BMD) was causally positively associated with left-hand grip strength (β = 0.017, *p*-value = 0.001), fat-free mass (FFM; right leg FFM, β = 0.014, *p*-value = 0.003; left arm FFM, β = 0.014, *p*-value = 0.005), but not walking pace. Higher hand grip strength was potentially causally associated with increased LS-BMD (right-hand grip strength, β = 0.318, *p*-value = 0.001; left-hand grip strength, β = 0.358, *p*-value = 3.97 × 10–4). In conclusion, osteoporosis may be a risk factor for sarcopenia-related traits and muscle strength may have a site-specific effect on BMD.

## INTRODUCTION

Sarcopenia and osteoporosis are the main clinical and socioeconomic burdens among many geriatric diseases [1, 2]. Both of them are closely related to motor function decline, falls and fractures, and lead to significant health and social costs [1, 3]. Sarcopenia, a complex multifactorial condition, is defined as a disease with low muscle mass plus low muscle strength and/or low physical performance [4]. Osteoporosis is one of the most common systemic bone diseases in humans. It is characterized by decreased bone density and bone mass, together with disruption of bone architecture, resulting in an increased risk of fragility fractures represents the main clinical consequence of the disease [5]. Studies have shown that sarcopenia can increase the risk of fractures, and the incidence of sarcopenia after fractures will also increase [6, 7]. About one third of postmenopausal women in the world suffer from osteoporosis, and at least half of them will experience a fragility fracture in their lifetime [8]. Fragility fractures are also closely related to increased morbidity and mortality, and can dramatically reduce the quality of life [9]. As sarcopenia and osteoporosis often appear at the same time, a new definition of geriatric disease has emerged, osteosarcopenia, which can be simply understood as a combination of these two diseases [10]. In the study of osteosarcopenia, it was found that there was a correlation between bone mineral density T-score values and handgrip strength [11].

Due to the adjacent surfaces of muscle and bone, the mechanical effects of muscle load on bone function, bones and muscles have been increasingly recognized as interacting tissues over the past decade [12–16]. This connection created the concept of a skeletal-muscle unit. More and more evidence showed that there is a link between sarcopenia and osteoporosis [17, 18]. Some studies have found that there is a positive correlation between muscle health indicators and either bone mass or density [19–21]. Additionally, studies using peripheral quantitative computed tomography (pQCT) have shown that bone size and strength are largely related to muscle size and to a lesser extent, are also related to muscle strength [22, 23]. Moreover, there is a clinical observation study that found sarcopenia increases the risk of 5-year mortality in patients with osteoporotic hip fractures [24], which suggests a link between sarcopenia and poor prognosis for osteoporotic fractures.

There are some possible mechanisms for the above-mentioned relationships [25]. The Mechanostat theory proposes that the direct mechanical stimulation caused by muscle contraction on bone can promote osteogenesis [26]. It is widely recognized that endocrine, genetic, developmental, lifestyle, including lack of physical activity, smoking, poor diet, and many other factors have dual effects on both muscle and bone quality [27–29]. For example, hormones, such as growth hormone, can promote both bone and muscle growth [27, 30, 31]. In addition, more exercise and higher activity levels can also significantly strengthen bone and muscle [28, 32]. Furthermore, muscle and bone may share some common genetic and developmental components [29]. Specific pathophysiological findings, such as fatty infiltration and musculoskeletal progenitor stem cells changes, have been revealed in both sarcopenia and osteoporosis [33–37]. Interesting, there are many common pathways for both sarcopenia and osteoporosis, such as sensitivity to decreased secretion of anabolic hormones, increased activity of inflammatory cytokines, and release of anabolic or catabolic molecules by skeletal muscle or bone cells (i.e., myokines and osteokine), which eventually lead to decreased physical activities [6, 19, 38, 39].

These factors and mechanisms associated with both sarcopenia and osteoporosis might influence the results of observational studies on the relationship between sarcopenia and osteoporosis. In addition, the causal relationships between sarcopenia and osteoporosis or fracture were still unclear. Fortunately, in recent years, due to the increasing attention to the bone and muscle health of the older adults, some genome-wide association studies (GWASs) have been completed and the genetic findings related to these characteristics have been revealed. Therefore, we used the bi-directional Mendelian randomization (MR) approach to assess the potential causal relationship between sarcopenia-related traits and osteoporosis/fracture. MR is a study design in which genetic variants can be used as instrumental variables (IVs) to infer the specific effect of exposure (such as sarcopenia) on outcome (such as osteoporosis) [40, 41]. Since the formation of gametes follows the Mendelian law, the genetic genes of parents are randomly assigned to the offspring. Therefore, genotype is likely to be independent of the factors that may confuse observational studies. MR approach can also distinguish the factors of symptoms and causes, which can avoid the reversal of causality [42]. Here, we undertook the bi-directional two-sample MR, which uses GWAS summary-level data [43, 44]. In two-sample MR, the IVs of the exposure and the outcome come from two independent GWAS. In the present study, the application of bi-directional MR design made our results more robust to confounding factors and reverse causation. In this way, if both sarcopenia-related traits and osteoporosis can be seen as causes to promote the occurrence of the other disease, we will not miss the causal relationship in either direction. In addition, we used GWASs data for osteoporosis-related traits (bone mineral density, BMD) from two independent populations as discovery samples and validation samples for analysis respectively, to clarify the causal relationship between sarcopenia-related traits and osteoporosis. According to the best of our knowledge, there is no similar MR study to explore the causal effects between sarcopenia-related traits and osteoporosis so far.

## RESULTS

### Influence of genetically predicted sarcopenia-related traits on osteoporosis

In the discovery cohort, the results did not show that the sarcopenia-related traits as exposure factors may have a potential causal relationship with Heel-BMD. We obtained 169, 155, 495, 491, 500, 507, 542, and 56 linkage disequilibrium (LD)-independent (*r^2^* < 0.001) IVs that achieved genome-wide significance level (*p* < 5 × 10^−8)^ for right-hand grip strength, left-hand grip strength, right leg fat-free mass (FFM), left leg FFM, right arm FFM, left arm FFM, whole body FFM, and walking pace, respectively. These IVs were used for analysis with Heel-BMD. Because the negative control analysis results showed that right-hand grip strength, left-hand grip strength, right leg FFM, left leg FFM, right arm FFM, left arm FFM, whole body FFM, and walking pace were not causally related to myopia, we believe that the selected IVs of exposures were appropriate (Supplementary Table 1). The heterogeneity test showed significant heterogeneity among selected IVs (*p* < 0.05, Supplementary Table 2). In consideration of this, the inverse-variance weighted (IVW) method with multiplicative random effects was used in the following MR analyses. As mentioned above, we used a Bonferroni corrected significance level of 0.00625 (0.05/8). IVW analysis showed no significant causal association between the genetically instrumented sarcopenia-related traits and Heel-BMD, as *p*-values did not reach the significance level (Supplementary Table 2). The MR-Egger analysis detected the existence of directional pleiotropy in IVs for appendicular FFM and whole body FFM (Supplementary Table 2). However, no significant causal relationship between the genetically instrumented sarcopenia-related traits and Heel-BMD was found after using the Mendelian Randomization Pleiotropy RESidual Sum and Outlier (MR-PRESSO) to adjust the directional pleiotropy (Supplementary Table 3). The same results were obtained by Robust Adjusted Profile Score (RAPS) (Supplementary Table 3). Based on the above results, the genetically predicted sarcopenia-related traits did not have a causal impact on Heel-BMD in the discovery cohort.

In the replication cohort, the results showed that only left and right hand grip strength as exposure factors could have positive causal correlations with LS-BMD. There were 159, 147, 455, 448, 461, 466, 493, and 56 LD-independent (*r^2^* < 0.001) single nucleotide polymorphisms (SNPs) achieving genome-wide significance level (*p* < 5 × 10^−8)^ that can be used as IVs for right-hand grip strength, left-hand grip strength, right leg FFM, left leg FFM, right arm FFM, left arm FFM, whole body FFM, and walking pace, respectively. These IVs were used for analysis with lumbar spine BMD (LS-BMD) and femoral neck BMD (FNK-BMD). Same as the discovery cohort, the negative control analysis results showed that the selected IVs were appropriate (Supplementary Table 1). IVW in a random-effects model was used according to the results of the heterogeneity test. IVW analysis showed both right-hand grip strength [β = 0.318, 95% confidence interval (CI) 0.138–0.498, *p*-value = 0.001; β in the results represents the effect value of IVs on outcomes] and left-hand grip strength (β = 0.358, 95% CI 0.16–0.556, *p*-value = 3.97 × 10^−4^) were causally positively associated with LS-BMD (Table 1). The MR-Egger analysis did not detect any directional pleiotropy for our selected IVs. Consistent with the IVW results, the estimates of Weighted median, MR-PRESSO, and RAPS identified a similar causal effect of hand grip strength on LS-BMD (Table 2). No significant causal relationship between the genetically instrumented hand grip strength and FNK-BMD was found. Other sarcopenia-related traits were not found to be causally associated with LS-BMD or FNK-BMD.

**Table 1:** Association of sarcopenia-related traits with FNK-BMD and LS-BMD using MR-Egger and IVW analysis.

**Exposures**	**Outcomes**	**No. of IVs**	**Heterogeneity test**	**MR Egger**		**IVW (random-effect model)**	
			**Cochran’s Q (*p*)**	**Intercept (*p*)**	***p***	**Estimates (95% CI)**	***p***
**Right hand grip strength**	**LS-BMD**	159	269.446 (<0.001)	0.003	0.529	0.318 (0.138, 0.498)	0.001^*^
**Left hand grip strength**	**LS-BMD**	147	273.941 (<0.001)	−0.003	0.574	0.358 (0.160, 0.556)	3.97E-04
**Right leg FFM**	**LS-BMD**	455	693.106 (<0.001)	−0.001	0.598	0.090 (0.002, 0.179)	0.045
**Left leg FFM**	**LS-BMD**	448	647.665 (<0.001)	0.000	0.998	0.103 (0.017, 0.190)	0.019
**Right arm FFM**	**LS-BMD**	461	750.518 (<0.001)	0.002	0.206	0.118 (0.021, 0.214)	0.017
**Left arm FFM**	**LS-BMD**	466	736.733 (<0.001)	0.000	0.88	0.099 (0.006, 0.192)	0.036
**Whole body FFM**	**LS-BMD**	493	756.673 (<0.001)	0.000	0.922	0.098 (0.012, 0.184)	0.025
**Walking pace**	**LS-BMD**	56	61.127 (0.265)	−0.002	0.725	−0.027 (-0.449, 0.395)	0.901
**Right hand grip strength**	**FNK-BMD**	159	314.781 (<0.001)	0.002	0.575	−0.021 (−0.216, 0.173)	0.829
**Left hand grip strength**	**FNK-BMD**	147	235.815 (<0.001)	−0.001	0.882	0.082 (−0.076, 0.240)	0.31
**Right leg FFM**	**FNK-BMD**	455	784.053 (<0.001)	0.002	0.119	−0.039 (−0.120, 0.042)	0.343
**Left leg FFM**	**FNK-BMD**	448	679.473 (<0.001)	0.002	0.062	−0.023 (-0.099, 0.053)	0.548
**Right arm FFM**	**FNK-BMD**	461	766.006 (<0.001)	0.001	0.277	−0.004 (−0.087, 0.080)	0.933
**Left arm FFM**	**FNK-BMD**	466	804.892 (<0.001)	0.001	0.363	0.002 (−0.082, 0.085)	0.967
**Whole body FFM**	**FNK-BMD**	493	870.233 (<0.001)	0.001	0.629	−0.02 (−0.099, 0.059)	0.614
**Walking pace**	**FNK-BMD**	56	48.029 (0.736)	0.007	0.145	0.119 (−0.129, 0.366)	0.348

^*^p < 0.00625. Abbreviations: MR: Mendelian randomization; IVW: inverse variance weighted; IVs: instrumental variables; CI: confidence interval; FFM: fat-free mass.

**Table 2:** Association of FNK-BMD and LS-BMD with sarcopenia-related traits using weighted median, RAPS and MR-PRESSO analysis.

**Exposures**	**Outcomes**	**No. of IVs**	**Weighted median**		**RAPS**		**MR-PRESSO**	
			**Estimates (95% CI)**	***p***	**Estimates (95% CI)**	***p***	**Estimates (95% CI)**	***p***
**Right hand grip strength**	**LS-BMD**	159	0.226 (0.014, 0.438)	0.037	0.273 (0.093, 0.452)	0.003^*^	0.291 (0.128, 0.455)	0.001^*^
**Left hand grip strength**	**LS-BMD**	147	0.398 (0.162, 0.634)	0.001^*^	0.325 (0.107, 0.544)	0.004^*^	0.333 (0.150, 0.517)	0.001^*^
**Right leg FFM**	**LS-BMD**	455	0.077 (−0.045, 0.199)	0.215	0.074 (−0.015, 0.163)	0.104	0.072 (−0.014, 0.158)	0.099
**Left leg FFM**	**LS-BMD**	448	0.079 (−0.007, 0.166)	0.072	0.079 (−0.007, 0.166)	0.072	0.099 (0.016, 0.183)	0.020
**Right arm FFM**	**LS-BMD**	461	0.085 (−0.010, 0.180)	0.080	0.085 (−0.010, 0.180)	0.080	0.107 (0.017, 0.197)	0.020
**Left arm FFM**	**LS-BMD**	466	0.116 (−0.012, 0.245)	0.076	0.085 (−0.010, 0.179)	0.079	0.088 (−0.002, 0.177)	0.056
**Whole body FFM**	**LS-BMD**	493	0.100 (−0.018, 0.218)	0.097	0.079 (−0.007, 0.165)	0.070	0.103 (0.020, 0.186)	0.015
**Walking pace**	**LS-BMD**	56	−0.027 (−0.449, 0.395)	0.901	−0.037 (−0.350, 0.277)	0.818		
**Right hand grip strength**	**FNK-BMD**	159	−0.021 (−0.216, 0.173)	0.829	−0.021 (−0.188, 0.146)	0.803	−0.036 (−0.19, 0.118)	0.650
**Left hand grip strength**	**FNK-BMD**	147	0.032 (−0.173, 0.236)	0.760	0.053 (−0.115, 0.221)	0.535	0.060 (−0.094, 0.214)	0.447
**Right leg FFM**	**FNK-BMD**	455	−0.057 (−0.135, 0.020)	0.146	−0.057 (−0.135, 0.020)	0.146	−0.060 (−0.132, 0.012)	0.102
**Left leg FFM**	**FNK-BMD**	448	−0.072 (−0.177, 0.034)	0.185	−0.037 (−0.113, 0.039)	0.344	−0.043 (−0.115, 0.030)	0.249
**Right arm FFM**	**FNK-BMD**	461	−0.039 (−0.144, 0.067)	0.475	−0.018 (−0.103, 0.066)	0.673	−0.051 (−0.129, 0.027)	0.200
**Left arm FFM**	**FNK-BMD**	466	−0.034 (−0.143, 0.075)	0.546	−0.020 (−0.099, 0.059)	0.614	−0.015 (−0.094, 0.064)	0.714
**Whole body FFM**	**FNK-BMD**	493	−0.074 (−0.175, 0.027)	0.151	−0.040 (−0.117, 0.038)	0.314	−0.049 (−0.12, 0.021)	0.172
**Walking pace**	**FNK-BMD**	56	0.165 (−0.196, 0.526)	0.371	0.130 (−0.131, 0.391)	0.330		

^*^p < 0.00625. Abbreviations: RAPS: robust adjusted profile score; IVs: instrumental variables; CI: confidence interval; FFM: fat-free mass.

After removal of the proxy SNPs, both left-hand grip strength and right-hand grip strength were still causally positively associated with LS-BMD in the estimates of all analysis approach significantly. Therefore, it can be determined that the genetically predicted grip strength have a causal effect on LS-BMD in the replication cohort. And the influence direction is positive, that is, greater grip strength corresponds to higher LS-BMD.

### Influence of genetically predicted osteoporosis on sarcopenia-related traits

In the discovery cohort, the results pointed to a positive causal association between Heel-BMD as an exposure factor and grip strength and FFM. We obtained 350 LD-independent (*r^2^* < 0.001) SNPs achieving genome-wide significance level (*p* < 5 × 10^−8^) that can be used as IVs for Heel-BMD. The negative control analysis results showed that the selected IVs were appropriate (Supplementary Table 1), and IVW in a random-effects model was used according to the heterogeneity test results. The MR-Egger analysis detected the existence of directional pleiotropy in IVs (Table 3). Though the IVW analysis showed no significant causal association between the genetically instrumented Heel-BMD and sarcopenia-related traits, the adjusted results of MR-PRESSO are more meaningful. The adjusted estimates showed Heel-BMD was causally positively associated with left-hand grip strength (β = 0.017, 95% CI 0.007–0.027, *p*-value = 0.001), right leg FFM (β = 0.014, 95% CI 0.005–0.023, *p*-value = 0.003) and left arm FFM (β = 0.014, 95% CI 0.004–0.023, *p*-value = 0.005) (Table 4). While the influence of genetically predicated Heel-BMD on right-hand grip strength (β = 0.014, 95% CI 0.004–0.024, *p*-value = 0.007), left leg FFM (β = 0.012, 95% CI 0.003–0.021, *p*-value = 0.009) and right arm FFM (β = 0.011, 95% CI 0.002–0.021, *p*-value = 0.023) evaluated by MR-PRESSO did not reach the significance level after Bonferroni correction, they all showed the same direction. The results of sensitivity analyses were consistent with MR-PRESSO, though some of them did not reach the corrected significance level. In general, it is reasonable to believe that genetically predicted Heel-BMD have a positive causal effect on the sarcopenia- related traits, including grip strength and FFM.

**Table 3:** Association of Heel-BMD with sarcopenia-related traits using MR-Egger and IVW analysis.

**Exposures**	**Outcomes**	**No. of IVs**	**Heterogeneity test**	**MR Egger**		**IVW (random-effect model)**	
			**Cochran’s Q (*p*)**	**Intercept**	***p***	**Estimates (95% CI)**	***p***
**Heel-BMD**	**Right hand grip strength**	350	1783.284 (<0.001)	−0.001	0.165	0.009 (−0.004, 0.022)	0.185
**Heel-BMD**	**Left hand grip strength**	350	1643.761 (<0.001)	−0.001	0.147	0.014 (0.001, 0.026)	0.038
**Heel-BMD**	**Right leg FFM**	350	5003.631 (<0.001)	−0.002	0.002	0.001 (−0.019, 0.020)	0.947
**Heel-BMD**	**Left leg FFM**	350	4878.473 (<0.001)	−0.002	0.002	−0.001 (−0.020, 0.019)	0.948
**Heel-BMD**	**Right arm FFM**	350	5256.588 (<0.001)	−0.002	0.001	0.005 (−0.014, 0.024)	0.604
**Heel-BMD**	**Left arm FFM**	350	5031.616 (<0.001)	0.001	0.001	0.006 (−0.013, 0.025)	0.556
**Heel-BMD**	**Whole body FFM**	350	6182.129 (<0.001)	−0.002	0.001	0.001 (−0.020, 0.021)	0.957
**Heel-BMD**	**Walking pace**	350	731.186 (<0.001)	0.000	0.585	−0.007 (−0.015, 0.000)	0.044

^*^p < 0.00625. Abbreviations: MR: Mendelian randomization; IVW: inverse variance weighted; IVs: instrumental variables; CI: confidence interval; FFM: fat-free mass.

**Table 4:** Association of Heel-BMD with sarcopenia-related traits using weighted median, RAPS and MR-PRESSO analysis.

**Exposures**	**Outcomes**	**No. of IVs**	**Weighted median**		**RAPS**		**MR-PRESSO**	
			**Estimates (95% CI)**	***p***	**Estimates (95% CI)**	***p***	**Estimates (95% CI)**	***p***
**Heel-BMD**	**Right hand grip strength**	350	0.008 (-0.004, 0.019)	0.177	0.012 (0.000, 0.024)	0.052	0.014 (0.004, 0.024)	0.007
**Heel-BMD**	**Left hand grip strength**	350	0.012 (0.001, 0.024)	0.037	0.017 (0.005, 0.029)	0.007	0.017 (0.007, 0.027)	0.001^*^
**Heel-BMD**	**Right leg FFM**	350	0.024 (0.013, 0.035)	1.42E-05^*^	0.016 (0.000, 0.032)	0.050	0.014 (0.005, 0.023)	0.003^*^
**Heel-BMD**	**Left leg FFM**	350	0.023 (0.012, 0.033)	2.03E-05^*^	0.014 (−0.002, 0.030)	0.084	0.012 (0.003, 0.021)	0.009
**Heel-BMD**	**Right arm FFM**	350	0.011 (0.000, 0.021)	0.040	0.02 (0.005, 0.035)	0.007	0.011 (0.002, 0.021)	0.023
**Heel-BMD**	**Left arm FFM**	350	0.013 (0.004, 0.022)	0.005^*^	0.02 (0.005, 0.035)	0.008	0.014 (0.004, 0.023)	0.005^*^
**Heel-BMD**	**Whole body FFM**	350	0.017 (0.007, 0.027)	0.001^*^	0.017 (0.000, 0.033)	0.045	0.008 (−0.002, 0.017)	0.113
**Heel-BMD**	**Walking pace**	350	−0.003 (−0.012, 0.005)	0.456	−0.007 (−0.014, 0.001)	0.071	−0.007 (−0.014, 0.000)	0.049

^*^p < 0.00625. Abbreviations: RAPS: robust adjusted profile score; IVs: instrumental variables; CI: confidence interval; FFM: fat-free mass.

In the replication cohort, the results of the analysis showed that LS-BMD and FNK-BMD, as exposure factors, were significantly positively correlated with all sarcopenia-related traits except walking pace. We obtained 22 LD-independent (*r*^2^ < 0.001) SNPs achieving genome-wide significance level (*p* < 5 × 10^−8)^ for LS-BMD and 21 for FNK-BMD. The negative control analysis results showed that the selected IVs were appropriate (Supplementary Table 1). The IVW in a random-effects model was used according to the heterogeneity test results. The MR-Egger analysis did not detect any directional pleiotropy for our selected IVs, supporting that the results of IVW were credible. Excluding the walking pace, the LS-BMD and FNK-BMD were significantly causally positively correlated with all other sarcopenia-related traits (Table 5). Consistent with the IVW results, the MR-PRESSO results confirmed that LS-BMD and FNK-BMD were causally positively associated with grip strength and FFM (Table 6). As for the results of RAPS, the causal association between FNK-BMD and right-hand grip strength did not reach a significance level. The causal association between FNK-BMD and both hand grip estimated by Weighted median were not significant. Other results of sensitivity analyses were consistent with the IVW method. Based on the above results, there is an obvious positive causal relationship between the genetically predicted LS-BMD and FNK-BMD and the sarcopenia-related traits (except walking pace). This potential causal association is consistent with the results of the analysis using Heel-BMD in the discovery cohort, highlighting that the reduction of BMDs is likely to lead to the reduction of muscle strength and lean body mass.

**Table 5:** Association of FNK-BMD and LS-BMD with sarcopenia-related traits using MR-Egger and IVW analysis.

**Exposures**	**Outcomes**	**No. of IVs**	**Heterogeneity test**	**MR Egger**		**IVW (random-effect model)**	
			**Cochran’s Q (*p*)**	**Intercept (*p*)**	***p***	**Estimates (95% CI)**	***p***
**LS-BMD**	**Right hand grip strength**	22	71.137 (<0.001)	0.004	0.134	0.037 (0.018, 0.056)	1.50 E-04^*^
**LS-BMD**	**Left hand grip strength**	22	60.094 (<0.001)	0.004	0.131	0.033 (0.015, 0.050)	2.11 E-04^*^
**LS-BMD**	**Right leg FFM**	22	159.391 (<0.001)	0.003	0.459	0.049 (0.024, 0.074)	1.15 E-04^*^
**LS-BMD**	**Left leg FFM**	22	155.980 (<0.001)	0.003	0.396	0.047 (0.022, 0.071)	1.90 E-04^*^
**LS-BMD**	**Right arm FFM**	22	124.291 (<0.001)	0.004	0.214	0.054 (0.034, 0.075)	3.04E-07^*^
**LS-BMD**	**Left arm FFM**	22	115.763 (<0.001)	0.004	0.161	0.053 (0.032, 0.073)	4.53E-07^*^
**LS-BMD**	**Whole body FFM**	22	162.964 (<0.001)	0.004	0.24	0.053 (0.029, 0.077)	1.60 E-05^*^
**LS-BMD**	**Walking pace**	22	49.715 (<0.001)	−0.001	0.751	−0.014 (−0.027, 0.000)	0.042
**FNK-BMD**	**Right hand grip strength**	21	82.603 (<0.001)	0.004	0.297	0.034 (0.010, 0.058)	0.005^*^
**FNK-BMD**	**Left hand grip strength**	21	70.021 (<0.001)	0.004	0.238	0.034 (0.012, 0.056)	0.002^*^
**FNK-BMD**	**Right leg FFM**	21	188.314 (<0.001)	0.011	0.029	0.051 (0.020, 0.083)	0.001^*^
**FNK-BMD**	**Left leg FFM**	21	180.373 (<0.001)	0.01	0.038	0.051 (0.020, 0.082)	0.001^*^
**FNK-BMD**	**Right arm FFM**	21	158.111 (<0.001)	0.009	0.041	0.064 (0.036, 0.091)	5.53E-06^*^
**FNK-BMD**	**Left arm FFM**	21	157.948 (<0.001)	0.009	0.045	0.064 (0.036, 0.092)	7.94E-06^*^
**FNK-BMD**	**Whole body FFM**	21	220.869 (<0.001)	0.011	0.037	0.064 (0.032, 0.097)	1.08 E-04^*^
**FNK-BMD**	**Walking pace**	21	27.197 (0.130)	0.001	0.542	−0.001 (−0.013, 0.010)	0.838

^*^p < 0.00625. Abbreviations: MR: Mendelian randomization; IVW: inverse variance weighted; IVs: instrumental variables; CI: confidence interval; FFM: fat-free mass.

**Table 6:** Association of FNK-BMD and LS-BMD with sarcopenia-related traits using weighted median, RAPS and MR-PRESSO analysis.

**Exposures**	**Outcomes**	**No. of IVs**	**Weighted median**		**RAPS**		**MR-PRESSO**	
			**Estimates (95% CI)**	***p***	**Estimates (95% CI)**	***p***	**Estimates (95% CI)**	***p***
**LS-BMD**	**Right hand grip strength**	22	0.044 (0.027, 0.062)	4.94E-07^*^	0.033 (0.013, 0.054)	0.001^*^	0.042 (0.027, 0.058)	4.30E-05^*^
**LS-BMD**	**Left hand grip strength**	22	0.030 (0.013, 0.047)	4.52E-04^*^	0.030 (0.011, 0.049)	0.002^*^	0.038 (0.021, 0.054)	1.96E-04^*^
**LS-BMD**	**Right leg FFM**	22	0.038 (0.022, 0.054)	2.48E-06^*^	0.045 (0.027, 0.062)	6.72E-07^*^	0.045 (0.032, 0.057)	1.26E-06^*^
**LS-BMD**	**Left leg FFM**	22	0.035 (0.019, 0.050)	1.03E-05^*^	0.041 (0.023, 0.060)	1.23E-05^*^	0.043 (0.030, 0.055)	3.45E-06^*^
**LS-BMD**	**Right arm FFM**	22	0.042 (0.027, 0.056)	2.20E-08^*^	0.044 (0.026, 0.062)	2.01E-06^*^	0.044 (0.034, 0.055)	2.02E-07^*^
**LS-BMD**	**Left arm FFM**	22	0.048 (0.034, 0.062)	2.74E-11^*^	0.042 (0.023, 0.062)	1.67E-05^*^	0.047 (0.035, 0.059)	5.21E-07^*^
**LS-BMD**	**Whole body FFM**	22	0.042 (0.027, 0.057)	4.48E-08^*^	0.041 (0.021, 0.062)	8.00E-05^*^	0.042 (0.030, 0.054)	2.29E-06^*^
**LS-BMD**	**Walking pace**	22	−0.012 (−0.027, 0.002)	0.100	−0.012 (−0.026, 0.002)	0.087		
**FNK-BMD**	**Right hand grip strength**	21	0.018 (−0.003, 0.039)	0.101	0.034 (0.005, 0.063)	0.021	0.036 (0.014, 0.058)	0.006^*^
**FNK-BMD**	**Left hand grip strength**	21	0.018 (−0.002, 0.038)	0.073	0.036 (0.010, 0.062)	0.006^*^	0.04 (0.019, 0.061)	0.002^*^
**FNK-BMD**	**Right leg FFM**	21	0.039 (0.019, 0.059)	1.50E-04^*^	0.052 (0.021, 0.083)	0.001^*^	0.053 (0.034, 0.071)	5.46E-05^*^
**FNK-BMD**	**Left leg FFM**	21	0.037 (0.018, 0.057)	1.93E-04^*^	0.052 (0.023, 0.082)	0.001^*^	0.047 (0.031, 0.064)	5.78E-05^*^
**FNK-BMD**	**Right arm FFM**	21	0.045 (0.025, 0.065)	9.57E-06^*^	0.060 (0.032, 0.089)	3.38E-05^*^	0.058 (0.038, 0.079)	5.29E-05^*^
**FNK-BMD**	**Left arm FFM**	21	0.053 (0.033, 0.072)	1.26E-07^*^	0.060 (0.030, 0.090)	8.09E-05^*^	0.059 (0.039, 0.079)	4.43E-05^*^
**FNK-BMD**	**Whole body FFM**	21	0.047 (0.027, 0.067)	5.81E-06^*^	0.060 (0.026, 0.093)	4.89E-04^*^	0.059 (0.039, 0.078)	5.38E-05^*^
**FNK-BMD**	**Walking pace**	21	0.002 (−0.013, 0.017)	0.774	0.000 (−0.012, 0.012)	0.966		

^*^p < 0.00625. Abbreviations: RAPS: robust adjusted profile score; IVs: instrumental variables; CI: confidence interval; FFM: fat-free mass.

### Influence of genetically predicted sarcopenia-related traits on fracture

We used the sarcopenia-related traits as exposure factors, and failed to find a potential causal relationship between them and fracture. 169, 155, 491, 487, 495, 505, 539, 56 LD-independent (*r^2^* < 0.001) SNPs achieved genome-wide significance level (*p* < 5 × 10^−8^) respectively for right-hand grip strength, left-hand grip strength, right leg FFM, left leg FFM, right arm FFM, left arm FFM, whole body FFM, walking pace that can assess the influence of sarcopenia-related traits on fracture resulting from simple fall. The negative control analysis results showed that the selected IVs were appropriate (Supplementary Table 1). IVW in a random-effects model was used according to the heterogeneity test results, and the MR-Egger analysis did not detect any directional pleiotropy. We did not detect a causal relationship between the genetically predicted sarcopenia-related traits on fracture (Supplementary Tables 4, 5). After removing the only proxy instrumental variant for whole body FFM, the results were almost unchanged. We still have not detected a potential causal relationship between genetically predicted sarcopenia-related traits and fracture.

## DISCUSSION

In this study, we used summary-level data to perform the bi-directional two-sample MR analysis and confirmed the causal associations between sarcopenia-related traits and osteoporosis. The results showed that genetically predicted BMDs at different skeletal sites were causally positively correlated to hand grip strength and FFM, but have no causal association with walking pace. In the other direction, we did not find any causal relationships of genetically predicted sarcopenia-related traits on Heel-BMD in the discovery cohort, but the results in the replication cohort showed that grip strength was causally positively correlated to LS-BMD. This suggested that, to a greater extent, osteoporosis may lead to sarcopenia. Additionally, we also found no causal relationship between sarcopenia-related traits and fracture in both discovery and replication cohorts. As far as we know, this is the first bi-directional MR study to assess the potential and reverse causal relations between sarcopenia-related traits and osteoporosis or fracture.

Previous studies have found that skeletal muscle mass and muscle strength are associated with BMD. Several previous traditional experiments showed that lower muscle mass was related to lower BMD, and might be a risk factor for fracture [45]. Another study among 2711 premenopausal women found that skeletal muscle mass was positively related to BMD, especially hip BMD [46]. A study performed in old men has also shown that there were positive relationships between lean mass and FNK-BMD [47]. Through the evaluation of 100 young men, the data also proved that BMD was positively correlated with the appendicular lean mass (ALM) index (ALM/ height^2^), and the ALM index was the strongest factor related to BMD in the young population [48]. However, in our MR analyses, we did not identify any significant causal relationship between genetically predicted FFM and BMDs. Dual energy X-ray absorptiometry (DXA) is the standard method [49]. Although nowadays DXA machines are widely used and available worldwide, due to its high cost and long measurement time, some lean body mass measurements with large sample size adopted bioelectrical impedance analysis (BIA) method, which is characterized by fast and simple measurement process. The FFM data in this article was measured using BIA, not DXA. So, the IVs related to lean mass may have some limitations in causality analysis. On the contrary, our results pointed out that genetically predicted BMDs were causally positively associated with ALM and whole body FFM. This suggested that BMD may affect lean body mass, which is different from the causal relationship direction found in the above studies. As mentioned above, various factors and mechanism may act on bones and muscles, thus affecting the results of observational studies. Moreover, even if muscle can affect BMD through some physical mechanisms or the release of biological factors, it does not mean that the causal direction at the gene level is that sarcopenia causes osteoporosis. Our results tended to show that BMD may be a causal influencing factor of lean body mass, rather than lean body mass can affect BMD directly. It is worth noting that although the original purpose of this study was to analyze the causal relationship between sarcopenia-related traits and osteoporosis, we used bone mineral density as the study phenotype of osteoporosis in our study, rather than directly using the qualitative trait of diagnosed osteoporosis itself. BMD is not a qualitative variable, but a quantitative variable which is the most power predictor of osteoporosis risk. Therefore, a lower BMD not only represents osteoporosis, but can also include the condition that bone mineral density decreases but does not reach the level of diagnosis of osteoporosis, such as osteopenia. As a result, not only osteoporosis, osteopenia may also lead to sarcopenia.

The previous study has shown that low grip strength was correlated to low BMD, and was a risk factor for osteoporosis in postmenopausal women [50]. Takahiro Tachiki et al. carried on an analysis in postmenopausal Japanese women found that muscle strength was associated with BMD at femoral neck and lumbar spine independently of muscle mass [51]. For adolescents, hand grip strength was positively correlated to the bone density health of both sexes [52]. Yingying Luo et al. used data from the National Health and Nutrition Examination Survey suggested that hand grip strength was positively associated with BMD of nonadjacent bones [53]. In this present study, we did not find grip strength could causally affect Heel-BMD in the discovery cohort, but found that grip strength as an exposure factor was causally positively correlated with LS-BMD in the replication cohort. The inconsistent results here can be attributed to the following reasons. Firstly, as we know, the DXA is regarded as the “gold standard” diagnostic technique for BMD measuring. But the Heel-BMD in the discovery cohort is quantitative ultrasound (QUS) of the heel calcaneus estimated BMD, which may not be a very accurate BMD value. In addition, a study has been conducted to examine shared/causal variants for the DXA-derived FNK-BMD and QUS of the heel calcaneus estimated BMD (eBMD), and found that the genetic correlation between FNK-BMD and eBMD was estimated to be 0.64. The author detected that most of SNPs or putative causal SNPs associated with eBMD were not associated with FNK-BMD [54]. This also shows that the analysis with eBMD is not equivalent to the analysis with DXA-derived BMDs. Therefore, in the replication cohort, the BMD measured by the DXA method may better explain the causal relationship between grip strength and BMD. Secondly, our results indicated that muscle strength had different effects on BMD at different skeletal sites. In the replication cohort, FNK-BMD (estimated by DXA) showed no causal association with the grip strength. This may be partially due to the different composition of bones (cortical and trabecular bone and the significant regional variation in bone microstructure) from different skeletal sites, which is determined by genetic factors [55].

As mentioned above, grip strength may causally affect LS-BMD significantly. In the other direction, although the effect values were smaller, our results also showed that genetically predicted BMDs in different parts, including Heel-BMD, LS-BMD, FNK-BMD were causally positively correlated to grip strength. In other words, BMD is also likely to have a causal impact on grip strength. At the genetic level, grip strength and BMD may be the causal factor mutually, thereby affecting the phenotype of each other, albeit to different degrees. This makes the relationship between grip strength and BMD more complicated. The reason may be as mentioned above, bone and skeletal muscle are affected by many common factors, and they also interact through some factors.

Our study did not detect causal effects in any direction between walking pace and BMD, nor sarcopenia-related traits on fracture. This indicated that declined lean body mass and strength indices might be the risk factors of fracture, but they probably increase the risk of fracture through some non-genetic ways. However, it should be noted that the GWAS data of fracture used in this study were various fractures caused by falling from standing height or lower. It is well known that the participants in the UK Biobank are relatively young and healthy, so the sample size of fractures is small in this study. In addition, the types of fractures are heterogeneous in GWAS, which may affect the results, too. Therefore, the absence of positive results here does not mean that sarcopenia-related traits are not one of the causes of fractures. Subsequent studies can try to complete GWAS for some homogeneous fractures, to discuss in detail the causal relationship of muscle mass and strength indices on fractures in different parts. Further research is also necessary to make a thorough inquiry about the possible impact path of sarcopenia on fracture risk.

Although this study used a bi-directional two-sample MR design that has some significant advantages, such as it is more resistant to confounding factors and robust to reverse causation, uses summary statistical data to get greater statistical power, and the direction of causality can be clarified, it still has some potential limitations that should be considered. First of all, in the discovery cohort, we used Heel-BMD to represent BMD. However, due to the limitations of its detection method, the estimation made by it may not be very reliable, although there was a considerable sample size. As mentioned above, the DXA is regarded as the “gold standard” diagnostic technique of measuring BMD, while the Heel-BMD is eBMD of the heel calcaneus, with which SNPs associated were not consistent with SNPs associated with DXA-derived BMDs. Secondly, sample size of fractures is small in GWAS and the types of fractures are heterogeneous here. This may lead to insufficient ability of this study to find the potential causal relationship between sarcopenia-related traits and fractures. Thirdly, we have to admit that there are some limitations of the key assumptions of MR, because it is difficult to ensure that there is not any confounder of the exposure-outcome relation or any potential pleiotropic effects. Fourth, population stratification might lead to bias in our estimate, even if all GWAS analysis for SNP-exposure association and SNP-outcome association were mainly obtained from European individuals, which could minimize the impact of population stratification. And the MRC-IEU displayed numbers of statistical methods to help ensure that the population structure has no significant influence on the traits [56, 57]. Fifth, we are inadequately equipped to perform sex-specific or age-specific analysis because we used summary-level data from which original individual measurements cannot be obtained. But regardless of sarcopenia or osteoporosis, the incidence of them both varies with age and gender. Sixth, also because we used summary-level data, the results were easily influenced by different GWAS quality control and selection standards. Because of the existence of these potential limitations mentioned above, we tried to use two different summary statistics datasets from two independent cohorts for analysis to derive a more reliable conclusion here in our study. Finally, because the analysis principle of MR is to infer causality from the genetic level, we can only get the potential causal relationship, but we can’t determine the specific biological pathway causing this causality. Biological evidence on how BMDs affect the sarcopenia-related traits in the absence of fracture remains to be revealed by other future studies.

## CONCLUSION

Our study found that BMD was causally positively associated with hand grip strength, lean mass, but not walking pace in both discovery and replication cohorts. And higher hand grip strength was causally associated with increased LS-BMD. In conclusion, decreased BMD may lead to decreased lean body mass in both discovery and replication cohorts, that is, osteoporosis may be the risk factor for sarcopenia-related traits. This study may contribute to monitoring the muscle health status of the older adults and preventing the occurrence or development of sarcopenia. Moreover, compared with the muscle mass approximation or physical performance, hand grip strength as a proxy of muscular fitness may be more suitable for studying the causal relationship between sarcopenia-related traits and osteoporosis, because we found that only grip strength as an exposure factor can causally affect LS-BMD. Besides, because grip strengths were only causally positively associated with LS-BMD in the replication cohort, further research is necessary to make a thorough inquiry about the site-specific causal effects of muscle strength or lean body mass on BMD.

## MATERIALS AND METHODS

### Data sources

GWAS summary data for this study were obtained from the UK Biobank (discovery cohort) and the GEnetic Factors for OSteoporosis (GEFOS; replication cohort) consortiums. The UK Biobank is a large-scale biomedical database and research resource, and contains in-depth genetic and health information from approximately half a million United Kingdom participants, aged between 40 and 69 years [58]. In this study, we used summary statistics from the MRC-IEU UK Biobank GWAS pipeline published in 2019 [59]. The GEFOS consortium (http://www.gefos.org) is a large international cooperation organization, involving many famous research groups. This study used GWAS summary statistics for LS-BMD and FNK-BMD in European ancestry from the general population published in 2015 [60].

### GWAS summary statistics of sarcopenia-related traits

#### Hand grip strength

Grip strength as a widely used proxy of muscular fitness, is a simple and non-invasive way to measure general muscle strength [61]. Considering the need for a stronger correlation with general muscle strength, absolute grip strength rather than relative grip strength (absolute handgrip strength/weight) is a more appropriate proxy because the former may have a higher correlation with muscle strength [62]. The GWAS summary statistics for hand grip strength were obtained from the UK Biobank, which includes 461,089 United Kingdom individuals for right-hand grip, and 461,026 individuals for left-hand grip strength [59]. The grip strength was measured using a calibrated grip-strength device adjusted for hand size [63], and each SNP was tested for association with hand grip strength, adjusting for age, age^2^, sex, sex × age, and sex × age^2^ [58].

#### Lean mass

Because muscle mass cannot be directly measured in usual, a reliable muscle mass approximation is needed. At present, lean mass is considered to be a valid measure of muscle mass [64], representing lipid-free soft tissue including muscle mass, body water, protein, glycerol, and soft tissue mineral mass [65]. The GWAS summary statistics for whole-body lean mass (WBLM; *N* = 454, 850), as well as ALM (left leg, *N* = 454,805; right leg, *N* = 454,835; left arm, *N* = 454,672; right arm, *N* = 454,753) from the UK Biobank were conducted on European populations, measured using BIA, and adjusted for age, age^2^, sex, sex × age, and sex × age^2^ [58]. ALM, as the most commonly used approximate index of muscle mass in sarcopenia research, is more appropriate compared with WBLM [66], because the latter contains other components of non-fat soft tissues, such as lungs, liver, or other organs, which may affect the measurement results [67]. We used both ALM and WBLM as sarcopenia-related traits to get more comprehensive results.

#### Walking pace

Because low physical performance is a characteristic of sarcopenia, the measurement of gait speed is also a significant diagnostic criterion of sarcopenia. According to the practical clinical definition and consensus diagnostic criteria for age-related sarcopenia developed by the European Working Group on Sarcopenia in Older People (EWGSOP), sarcopenia can be diagnosed only when low muscle mass plus low muscle strength and/or low physical performance (gait speed ≤0.8m/s) are presented [68]. Genetic predictors of walking pace were assessed using the summary statistics from the UK Biobank, which includes 459,915 individuals of European ancestry [59].

### GWAS summary statistics of osteoporosis-related traits

#### Heel-BMD

The Heel-BMD is QUS of the heel calcaneus eBMD. This method is quick, safe, and relatively inexpensive, so it’s cost-effective for GWASs with large samples of individuals [54]. We used the GWAS summary statistics for Heel-BMD from the UK Biobank. 265,627 individuals of European ancestry identified SNPs associated with Heel-BMD that were measured by QUS [59].

#### LS-BMD and FNK-BMD

Although BMD can be measured in many ways, the DXA is regarded as the “gold standard” diagnostic technique [69]. Genetic predictors of DXA-derived BMD were obtained from the summary statistics of the GEFOS consortium. Femoral neck and lumbar spine are common skeletal sites where osteoporotic fractures often occur [60]. The genetic variants associated with FNK- BMD and LS-BMD we used here were identified by a large meta-analysis, performed by GEFOS consortium in 32,735 and 28,498 subjects of European ancestry respectively [60]. It involves summary statistics for approximately 10 million SNPs. The genetic association of each SNP with BMD tested was adjusted for age, age^2^, sex, and weight.

#### GWAS summary statistics of fracture

Considering that a major purpose of this study is to explore the potential causal relationship between sarcopenia-related traits and fracture, we chose the summary-level genetic data for fracture resulting from simple fall from the UK Biobank. The sample size was 43,883, including 26,126 fracture cases and 17,757 controls [59]. A simple fall is defined as falling from standing height or lower, including tripping and falling over, or falling from a stool or chair that you are sitting on. But falls from anything higher such as stairs and ladders, or from standing on a stool or chair are not simple falls. The fractures here are heterogeneous, including various fracture types in different body parts.

#### Selection and validation of IVs

In MR studies, the independent genetic variants used as IVs must satisfy the three key assumptions [70, 71]: (1) the IVs are strongly associated with the exposure; (2) the IVs have no pleiotropic associations with any known confounders; (3) the IVs have no association with the outcome except possibly through their association with the exposure. The latter two assumptions are considered to be independent of pleiotropy. To satisfy the first assumption of MR, we chose SNPs without linkage disequilibrium (*r^2^* < 0.001) that were strongly associated with the exposure factors, which achieved genome-wide significance level (*p* < 5 × 10^−8^). For those SNPs that cannot find the corresponding effect estimates in the outcome GWAS summary statistics, we used the SNPs that highly correlated (*r^2^* > 0.8) with them as proxies.

To evaluate whether the SNPs we selected above can be used as appropriate IVs, we performed a pleiotropy test to assess whether horizontal pleiotropy exists in selected SNPs by the MR-Egger method. In this method, if the intercept deviates from the origin, it illustrates that the IVs exist potential pleiotropic effects, which is shown by the *p*-value of the intercept term being less than 0.05. On the contrary, if the *p*-value of the intercept term is not less than 0.05, there is no evidence for horizontal pleiotropy across the selected IVs.

### Statistical analysis

In this study, we conducted a bi-directional MR study of sarcopenia-related traits and osteoporosis-related traits to explore the potential causal effects between sarcopenia and osteoporosis. In addition, we also used the sarcopenia-related traits as exposure factors, and the fracture as outcome to explore the potential causal effects of sarcopenia-related traits on fracture.

We applied the IVW method with multiplicative random effects as the primary approach to estimate the causal effect between exposure and outcome, which was considered the most reliable indicator if there was no evidence of directional pleiotropy in the selected IVs (*p* for MR-Egger intercept >0.05). The causal effect estimate of each SNP in MR was calculated as its corresponding outcome effect size divided by exposure effect size. Cochran’s *Q* test was conducted to estimate whether there was a high heterogeneity among the selected IVs [72]. If the heterogeneity is not significant, the fixed-effects model can be used, otherwise, the IVW method with multiplicative random effects is suitable. Considering the situation of multiple testing, we adopted a Bonferroni method to correct the significance level, using a stricter *p*-value threshold of 0.05/*n* (*n* represents the number of independent hypotheses).

### Sensitivity analysis

To further ensure the validity of the MR causal effect estimation, we conducted several sensitivity analyses.

Firstly, as mentioned above, we used the MR-Egger method to detect the potential pleiotropy of IVS [73], and the pleiotropy-corrected causal effect could derive from the estimate for MR-Egger regression slope [73]. The test efficiency of this method is low, so, in this study we only used the MR-Egger method to detect the pleiotropic effects, not to evaluate causal effects.

Secondly, a previous study proved that the weighted median method provides some distinct advantages over the MR-Egger approach, for example, its causal effect detection capability is higher, with lower type I error [74]. The weighted median approach is robust to generate correct estimates, which is consistent even up to 50% of the SNPs are invalid IVs [74]. Therefore, we also used the weighted median method as a complement for sensitivity analysis to assess the robustness of the MR estimates.

Thirdly, due to the some weak IVs that might be contained in the analyses, we performed a RAPS analysis to make our result more reliable. Generally speaking, the MR-RAPS method can produce a robust inference when there are many weak instruments in MR analysis.

Fourthly, we also used MR-PRESSO as a statistical procedure to identify and remove possible pleiotropic IVs. This method assumes that at least 50% of the SNPs are valid SNPs [75], and can detect pleiotropy by assessing outliers among the selected SNPs which contribute to the MR estimate. MR-PRESSO can also provide adjusted estimates by removing outliers and test the significant difference of causal effect estimation before and after the correction of outliers. In this study, if there was horizontal pleiotropy, the adjusted estimates obtained by MR-PRESSO were regarded as the main indicator of causal effect estimation.

Moreover, we removed the proxy SNPs for repeated analysis to eliminate the interference of proxy SNPs on MR analysis.

### Negative control

Because there is no evidence showing that sarcopenia-related traits or osteoporosis are causally associated with myopia (short sight), we chose myopia as negative control for our analysis to further prove the validity of the IVs we selected above. Summary-level genetic data for myopia (both eyes) were obtained from the UK Biobank cohort, including 29,317 individuals of European ancestry [59].

All statistical analyses were implemented by the Two-sample MR package in the R software environment [76, 77].

## Supplementary Materials

Supplementary Tables
