# Supplementary material for: A bi-directional Mendelian randomization study of the sarcopenia-related traits and osteoporosis
Source: Aging (Albany NY). 2022 Jul 2;14(14):5681–98. doi: 10.18632/aging.204145 (PMC9365559; doi:10.18632/aging.204145)
Supplement: Supplementary Tables [file aging-14-204145-s001.pdf]

SUPPLEMENTARY TABLES

Supplementary Table 1. Negative control analysis.

Exposures	Outcomes	No. of IVs	IVW		Weighted median		RAPS	
			Estimates	*p*	Estimates	*p*	Estimates	*p*
**Right hand grip strength**	**Myopia**	169	−0.006	0.747	−0.026	0.356	−0.009	0.655
**Left hand grip strength**	**Myopia**	155	−0.011	0.576	−0.022	0.440	−0.015	0.476
**Right leg FFM**	**Myopia**	495	0.006	0.571	−0.006	0.705	0.002	0.848
**Left leg FFM**	**Myopia**	491	0.008	0.449	−0.013	0.408	0.004	0.692
**Right arm FFM**	**Myopia**	501	2.81E-05	0.998	−0.019	0.245	−0.001	0.936
**Left arm FFM**	**Myopia**	507	−0.004	0.682	−0.019	0.259	−0.004	0.705
**Whole body FFM**	**Myopia**	542	−0.006	0.523	−0.018	0.259	−0.008	0.423
**Walking pace**	**Myopia**	56	−0.029	0.494	−0.006	0.924	−0.026	0.568
**Heel-BMD**	**Myopia**	348	0.007	0.172	0.003	0.726	0.004	0.428
**LS-BMD**	**Myopia**	22	0.002	0.823	−0.004	0.759	0.001	0.948
**FNK-BMD**	**Myopia**	21	0.030	0.054	0.030	0.357	0.026	0.154

Abbreviations: IVW: inverse variance weighted; RAPS: robust adjusted profile score; IVs: instrumental variables; FFM: fat-free mass.

Supplementary Table 2. Association of sarcopenia-related traits with Heel-BMD using MR-Egger and IVW analysis.

Exposures	Outcomes	No. of IVs	Heterogeneity test	MR Egger		IVW (random-effect model)	
			Cochran's Q (*p*)	Intercept	*p*	Estimates (95% CI)	*p*
**Right hand grip strength**	**Heel-BMD**	169	1554.446 (<0.001)	0.003	0.226	−0.085 (−0.200, 0.029)	0.143
**Left hand grip strength**	**Heel-BMD**	155	1293.498 (<0.001)	0.003	0.688	−0.079 (−0.193, 0.035)	0.174
**Right leg FFM**	**Heel-BMD**	495	4123.616 (<0.001)	0.003	0.001	−0.002 (−0.059, 0.054)	0.934
**Left leg FFM**	**Heel-BMD**	491	4009.255 (<0.001)	0.003	0.002	−0.006 (−0.062, 0.050)	0.846
**Right arm FFM**	**Heel-BMD**	500	5091.533 (<0.001)	0.004	0.001	−0.023 (−0.089, 0.043)	0.502
**Left arm FFM**	**Heel-BMD**	507	4345.443 (<0.001)	0.003	0.005	0.001 (−0.059, 0.060)	0.983
**Whole body FFM**	**Heel-BMD**	542	4753.547 (<0.001)	0.003	0.001	−0.032 (−0.088, 0.024)	0.258
**Walking pace**	**Heel-BMD**	56	210.402 (<0.001)	−0.002	0.574	−0.182 (−0.344, −0.019)	0.029

$^{*}p < 0.00625$. Abbreviations: MR: mendelian randomization; IVW: inverse variance weighted; IVs: instrumental variables; CI: confidence interval; FFM: fat-free mass.

Supplementary Table 3. Association of sarcopenia-related traits with Heel-BMD using weighted median, RAPS and MR-PRESSO analysis.

Exposures	Outcomes	No. of IVs	Weighted median		RAPS		MR-PRESSO	
			Estimates (95% CI)	*p*	Estimates (95% CI)	*p*	Estimates (95% CI)	*p*
Right hand grip strength	Heel-BMD	169	−0.096 (−0.174, −0.017)	0.017	−0.070 (−0.219, 0.079)	0.359	−0.095 (−0.164, −0.025)	0.009
Left hand grip strength	Heel-BMD	155	−0.069 (−0.149, 0.012)	0.096	−0.065 (−0.231, 0.101)	0.443	−0.068 (−0.145, 0.008)	0.084
Right leg FFM	Heel-BMD	495	−0.028 (−0.073, 0.018)	0.232	−0.046 (−0.108, 0.017)	0.151	0.000 (−0.040, 0.040)	1.000
Left leg FFM	Heel-BMD	491	−0.008 (−0.052, 0.036)	0.723	−0.008 (−0.052, 0.036)	0.723	0.008 (−0.03, 0.047)	0.674
Right arm FFM	Heel-BMD	500	−0.073 (−0.123, −0.024)	0.003*	−0.082 (−0.155, -0.008)	0.029	−0.027 (−0.069, 0.014)	0.194
Left arm FFM	Heel-BMD	507	−0.008 (−0.052, 0.036)	0.727	−0.021 (−0.092, 0.050)	0.557	0.011 (−0.029, 0.051)	0.580
Whole body FFM	Heel-BMD	542	−0.067 (−0.112, −0.021)	0.004*	−0.057 (−0.120, 0.007)	0.079	−0.019 (−0.056, 0.019)	0.334
Walking pace	Heel-BMD	56	−0.125 (−0.258, 0.009)	0.068	−0.155 (−0.293, −0.018)	0.027	−0.149 (−0.257, −0.040)	0.010

**p* < 0.00625. Abbreviations: RAPS: robust adjusted profile score; IVs: instrumental variables; CI: confidence interval; FFM: fat-free mass.

Supplementary Table 4. Association of sarcopenia-related traits with fracture resulting from simple fall using MR-Egger and IVW analysis.

Exposures	Outcomes	No. of IVs	Heterogeneity test	MR Egger		IVW (random-effect model)	
			Cochran's Q (*p*)	Intercept	*p*	Estimates (95% CI)	*p*
Right hand grip strength	Fracture	169	175.436 (0.331)	0.000	0.805	−0.051 (−0.099, −0.003)	0.037
Left hand grip strength	Fracture	155	183.406 (0.053)	−0.001	0.444	−0.045 (−0.099, 0.008)	0.099
Right leg FFM	Fracture	491	565.085 (0.011)	0.000	0.391	0.010 (−0.016, 0.037)	0.448
Left leg FFM	Fracture	487	554.813 (0.017)	0.000	0.859	0.013 (−0.013, 0.040)	0.317
Right arm FFM	Fracture	495	605.811 (0.000)	−0.001	0.287	−0.001 (−0.030, 0.028)	0.941
Left arm FFM	Fracture	505	599.074 (0.002)	0.000	0.552	−0.008 (−0.036, 0.019)	0.559
Whole body FFM	Fracture	539	641.495 (0.001)	0.000	0.272	−0.001 (−0.027, 0.025)	0.921
Walking pace	Fracture	56	74.334 (0.042)	0.001	0.728	−0.134 (−0.255, −0.013)	0.030

**p* < 0.00625. Abbreviations: MR: mendelian randomization; IVW: inverse variance weighted; IVs: instrumental variables; CI: confidence interval; FFM: fat-free mass.

Supplementary Table 5. Association of sarcopenia-related traits with fracture resulting from simple fall using weighted median, RAPS and MR-PRESSO analysis.

Exposures	Outcomes	No. of IVs	Weighted median		RAPS		MR-PRESSO	
			Estimates (95% CI)	*p*	Estimates (95% CI)	*p*	Estimates (95% CI)	*p*
Right hand grip strength	Fracture	169	−0.05 (−0.123, 0.023)	0.178	−0.043 (−0.094, 0.007)	0.095		
Left hand grip strength	Fracture	155	−0.047 (−0.122, 0.027)	0.214	−0.032 (−0.087, 0.024)	0.264		
Right leg FFM	Fracture	491	0.001 (−0.041, 0.042)	0.977	0.013 (−0.015, 0.040)	0.368		
Left leg FFM	Fracture	487	0.010 (−0.031, 0.051)	0.640	0.015 (−0.012, 0.042)	0.277		
Right arm FFM	Fracture	495	0.000 (−0.041, 0.041)	0.995	0.003 (−0.028, 0.033)	0.858	0.001 (−0.028, 0.029)	0.966
Left arm FFM	Fracture	505	−0.004 (−0.044, 0.036)	0.861	−0.006 (−0.035, 0.023)	0.680	−0.007 (−0.034, 0.021)	0.637
Whole body FFM	Fracture	539	0.001 (−0.037, 0.038)	0.969	0.001 (−0.025, 0.028)	0.916	0.000 (−0.025, 0.026)	0.992
Walking pace	Fracture	56	−0.216 (−0.374, −0.057)	0.008	−0.148 (−0.271, −0.025)	0.019		

**p* < 0.00625. Abbreviations: RAPS: robust adjusted profile score; IVs: instrumental variables; CI: confidence interval; FFM: fat-free mass.
